# One-Step Extraction of Olive Phenols from Aqueous Solution Using β-Cyclodextrin in the Solid State, a Simple Eco-Friendly Method Providing Photochemical Stability to the Extracts

**DOI:** 10.3390/molecules26154463

**Published:** 2021-07-24

**Authors:** Aurélia Malapert, Emmanuelle Reboul, Olivier Dangles, Alain Thiéry, N’nabinty Sylla, Valérie Tomao

**Affiliations:** 1 Avignon University, INRAE, UMR408 MicroNut Team, 84000 Avignon, France; aurelia.malapert@univ-avignon.fr (A.M.); olivier.dangles@univ-avignon.fr (O.D.); 2 Aix-Marseille University, INRAE, INSERM, C2VN, Marseille, France; emmanuelle.reboul@univ-amu.fr; 3 Aix-Marseille Université, Avignon University, CNRS, IRD, IMBE UMR 7263, 13000 Marseille, France; alain.thiery@imbe.fr; 4 Université Mohamed V de Rabat, BP 8007, Rabat 10000, Morocco; nabinty62@yahoo.fr

**Keywords:** hydroxytyrosol, tyrosol, β-cyclodextrin, extraction

## Abstract

The extraction of phenolic compounds from olive mill wastes is important, not only to avoid environmental damages, but also because of the intrinsic value of those biophenols, well-known for their high antioxidant potential and health benefits. This study focuses on tyrosol (Tyr) and hydroxytyrosol (HT), two of the main phenolic compounds found in olive mill wastes. A new, simple, and eco-friendly extraction process for the removal of phenolic compounds from aqueous solutions using native β-cyclodextrin (β-CD) in the solid state has been developed. Several β-CD/biophenol molar ratios and biophenol concentrations were investigated, in order to maintain β-CD mostly in the solid state while optimizing the extraction yield and the loading capacity of the sorbent. The extraction efficiencies of Tyr and HT were up to 61%, with a total solid recovery higher than 90% using an initial concentration of 100 mM biophenol and 10 molar equivalents of β-CD. The photochemical stability of the complexes thus obtained was estimated from ∆E*ab curve vs. illumination time. The results obtained showed that the phenols encapsulated into solid β-CD are protected against photodegradation. The powder obtained could be directly developed as a safe-grade food supplement. This simple eco-friendly process could be used for extracting valuable biophenols from olive mill wastewater.

## 1. Introduction

In the Mediterranean basin, two of the main agricultural wastes are olive mill wastewaters (OMWW) and olive pomace, both of which are associated with olive oil production [1]. As much as 98% of olive phenolic compounds remain in wastes after olive mill processing and cause environmental damages upon spreading in fields [2]. The European Protection Agency (EPA), in collaboration with organizations, has developed regulations and laws restricting the phenolic content of wastewaters [3].

Previous studies have focused on decreasing the pollutant character of olive mill effluents by removing phenolic compounds. Iboukhoulef et al. [4] succeeded in reducing the phenolic content of OMWW by more than 60% in 65 min. This was accomplished by using an oxidative process based on Cu^I^ and hydrogen peroxide (Fenton reaction). Alternatively, an optimized ozonolytic process allowed the removal of more than 80% of phenolic compounds from wastewaters [5]. On the other hand, physical techniques, especially filtration methods, are commonly used for cleaning water effluents. For instance, Comandini et al. [6] removed phenolic compounds from OMWW by performing ultrafiltration followed by a reverse osmosis step. The ultrafiltration step removed about 40% of the phenolic compounds from the initial OMWW, then reverse osmosis yielded a phenolic-free permeate and a phenolic-rich concentrate. Other filtration methods rest on adsorption technologies. Aly et al. [7] successively used columns of gravel, fine sand, and a zeolite/acidified cotton mixture for removing pollutants from OMWW. Final steps with activated charcoal and lime led to safe effluents that could be used for irrigation. Abdelkreem [8] dried the olive mill pomace to turn it into an adsorbent that could be used for removing 85% of the phenolic compounds from OMWW. Using granular activated carbon (GAC), Aliakbarian et al. [9] removed 96% of phenolic compounds from OMWW and produced a clean water sample. Ena et al. [10] compared the ability of GAC and Azolla to remove phenolic compounds from OMWW. GAC showed higher adsorption and desorption capacities than Azolla, and the profile of the phenolic compounds retained was different according to the adsorbent used. While the total phenolic content was more than twice as high in the Azolla treated OMWW, HT was more abundant in the GAC-treated OMWW.

Apart from their polluting character, olive mill wastes have emerged as a valuable and economic source of natural phenolic compounds known for their diverse biological activities, including antioxidant, anti-inflammatory, and antimicrobial properties. Among them, HT is considered to be one of the most interesting phenolic compounds in olive oil and its by-products [11].

β-CD is a cyclic oligosaccharide composed of seven D-glucopyranose units connected by α-1,4 bonds. β-CD is “Generally Recognized as Safe” (GRAS) by the European Food Safety Agency (EFSA), which has defined an acceptable daily intake for β-CD of 5 mg/kg of body weight [12]. Due to its relatively nonpolar cavity and its hydrophilic outer surface, β-CD has a well-known ability to form inclusion complexes with a wide range of valuable compounds in aqueous solution, and is nowadays commonly used as a part of formulations for protecting or enhancing the solubility of guest compounds. β-CD also finds many applications in food processing for controlling flavor release or for reducing a bitter taste, for example. Complex formation depends on complementary sizes and the development of stabilizing molecular interactions between host and guest [13].

β-CD is also able to encapsulate phenolic compounds in an aqueous solution [14] and this property could therefore be used for reducing their bitter taste [15]. Moreover, López-García et al. [16] showed that β-CD could increase the antioxidant activity of HT by providing photoprotection. Host-guest complexes can also be formed in the solid state for convenient handling and storage. Spray- and freeze-drying processes are commonly used, and first require mixing β-CD and phenol in an aqueous solution to form the complexes. Over the last 10 years, works on phenolic compounds from olive leaves, [17] Melissa Officinalis leaves, [18] and blueberry juice [19] have been reported. Hundre et al. also prepared β-CD-vanillin complexes by spray drying, freeze drying, or by a combination of both, and concluded that the method selected has an impact on the external shape and thermal stability of the powder [20]. Other methods for preparing complexes in the solid state are the classical co-precipitation procedure, the paste technique, (or kneading, which consists in mixing the host and guest with a small amount of water), and solid phase complexation using a high level of mechanical energy to co-grind or extrude the complexes. Only a few studies have focused on the complexes of HT and Tyr with β-CD in the solid state. For instance, Garcia-Padial et al. [21] prepared the solid Tyr-β-CD complex by co-evaporation and kneading.

In this study, a new, simple, and eco-friendly process was developed for the one-step extraction of olive phenolic compounds from an aqueous solution by directly forming a solid-state complex with β-CD. The conditions were optimized in terms of phenol and β-CD concentration and molar ratio.

## 2. Results and discussion

### 2.1. Influence of the β-CD/Phenol Molar Ratio

**Figure 1:**
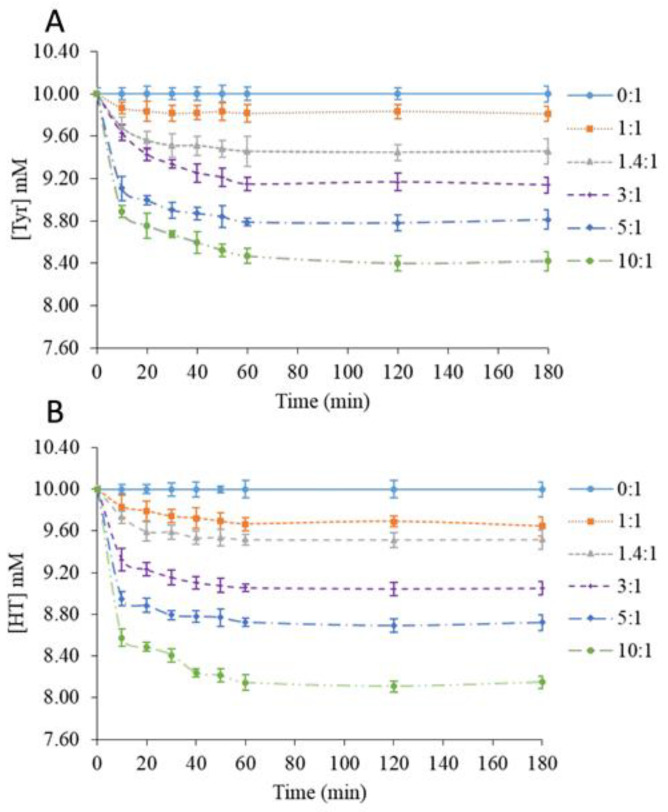
Time-dependence of tyrosol (**A**) and hydroxytyrosol (**B**) concentration according to the β-CD/phenol molar ratio.

Biophenols were extracted from 10 mM biophenol solutions containing various β-CD amounts over 3 h. The Tyr or HT concentration in the supernatant was plotted as a function of time (Figure 1A,B).

The studies of solid β-CD as a biophenol-extracting agent led to similar kinetics for Tyr and HT. Compared to standard solutions without β-CD (0:1), adding β-CD caused a fast decrease in the remaining biophenol concentration in the supernatant, which was complete after 60 min. This phenomenon could be explained by the fast adsorption of the phenols onto the solid to reach the equilibrium state [22,23]. Our observations were in agreement with the literature [24,25,26]. The β-CD/phenol molar ratios of 1:1 and 1.4:1 corresponded to β-CD below or at its solubility limit (16 g/L at 20 °C), and thus did not allow the recovery of powders. However, shifts in the phenols’ absorption bands consistent with the formation of inclusion complexes in a solution were observed. At higher molar ratios, solid recovery was possible. Wang et al. [27] studied the adsorption of phenols from wastewaters onto cross-linked β-CD-diphenylmethane diisocyanate polymer particles. The kinetics observed were similar with Tyr and HT.

As shown in Table 1, the solid recovery increased with the β-CD/Tyr molar ratio from 22.2% to 75.4% of the initial solid quantity (β-CD + Tyr). After correcting for the amount of solubilized β-CD, the values increased from 69.2% to 89.5%. For comparison, using freeze-drying, Jantarat et al. [28] obtained a 95.7% yield for curcumin encapsulated into a β*-*CD derivative. The extraction efficiency was calculated from the biophenol concentration in the rinsed MeOH fraction compared to the initial content. Results were in agreement with the kinetic data, showing an enhancement of the extraction efficiency at higher β-CD/Tyr molar ratios. Kumar et al. [29] proposed that a higher adsorbent amount, although it offers a larger number of available binding sites, could generate partial aggregation. The loading efficiency (Qe), which decreased with the β-CD quantity, seemed to be consistent with this view.

The same analyses were carried out with the β-CD-HT powder and gave similar data (Table 2).

Hence, it can be suggested that solid β-CD does not express selectivity in its extraction of Tyr or HT despite the higher affinity of the former for β-CD in aqueous solution, which is reflected by a ca. 10 times higher binding constant [21]. This is an indication that extraction does not proceed by the encapsulation of the phenols inside the β-CD cavity (as in aqueous solution) but rather by adsorption on the macrocycle’s surface, probably by hydrogen bonding. In that view, it may be significant that HT (2 phenolic OH groups, vs. only 1 for Tyr) tends to be slightly more efficiently extracted.

### 2.2. Influence of the Biophenol Concentration

**Figure 2:**
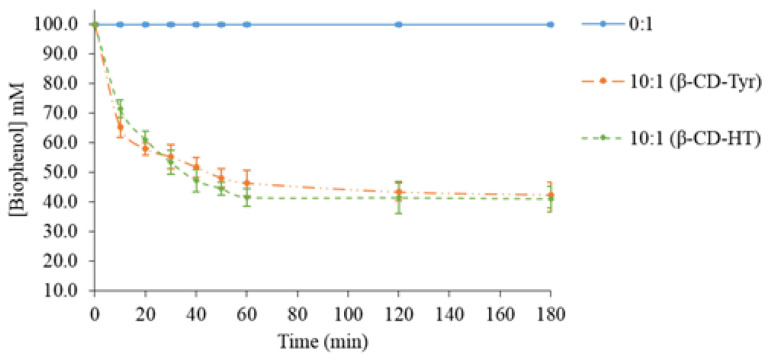
Time-dependence of the biophenol concentration in a concentrated aqueous solution according to the β-CD/phenol molar ratio of 10:1.

According to the preceding results, the highest extraction efficiency was obtained with 10 molar β-CD equivalents. This condition was further tested with concentrated solutions of olive phenols (100 mM) (Figure 2). 

As expected, the biophenol concentration in the aqueous phase decreased when the β-CD amount increased. For the same β-CD/biophenol molar ratio, this decrease was significantly higher than in dilute biophenol solutions (10 mM, Figure 1A,B). Table 3 presents the powder analysis.

Working with concentrated solutions of phenols permitted much better extraction yields (over 60%) to be reached. The loading efficiency Qe was still higher than in any experiment starting from a 10 mM Tyr solution. The assays on a concentrated HT solution gave almost identical results. 

### 2.3. Photochemical Stability

**Figure 3:**
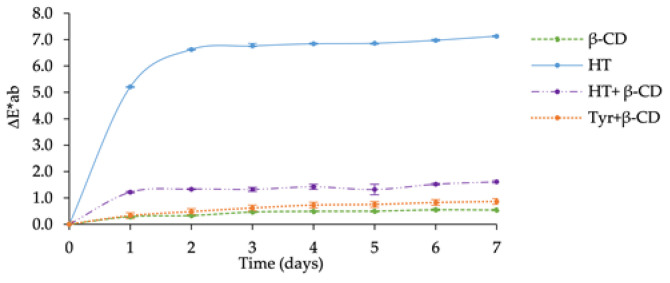
**∆**E*ab values of β-CD, HT, Tyr, HT+β-CD, Tyr+β-CD during photochemical degradation.

The photochemical stability of the phenol-β-CD complexes was assessed by plotting ∆E*ab vs. illumination time in comparison with the free phenols (Figure 3).

The results clearly showed significant differences between the kinetic profiles. HT exhibited a higher ΔE*ab value than HT+β-CD over time. The high photodegradation rate observed for free HT is in accordance with the sensitivity of catechols to oxidation. Interestingly, the solid matrix of β-CD strongly enhances the photostability of HT. The kinetic curves for Tyr and Tyr+β-CD exhibited similar trends with lower ∆E*ab values (consistent with Tyr being less oxidizable than HT). β-CD protects both phenols against photodegradation in the solid state. By providing a physical barrier between individual phenol molecules, β-CD possibly inhibits the photo-induced polymerization of phenols into brown products.

### 2.4. Scanning Electron Microscopy (SEM)

**Figure 4:**
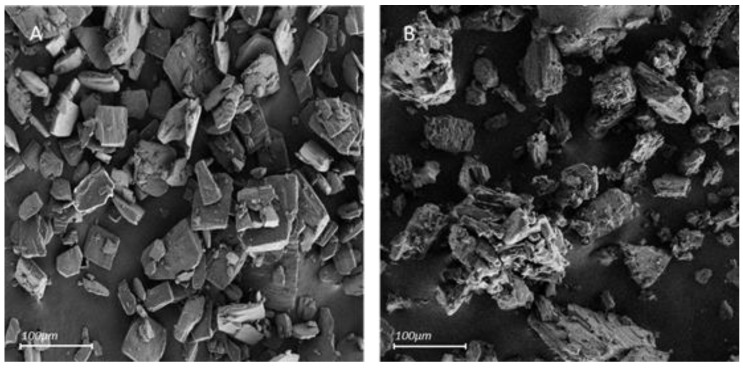
Scanning electron micrographs of β-CD (**A**) and the powder recovered after extraction of Tyr from a 100 mM aqueous solution (10:1 β-CD/Tyr molar ratio) (**B**) (Magnification: ×140).

The following SEM micrographs compare the external shapes of pure β-CD and β-CD + Tyr particles (Figure 4).

Pure β-CD has a microsized, angular shape, producing geometrical structures. The solid β-CD-Tyr particles also display the microsized morphology. However, the external surface is very irregular and striated compared to pure β-CD. This characteristic could be caused by Tyr particles deposited onto the β-CD particles.

In conclusion, this simple process potentially provides an efficient and sustainable method to extract and stabilize olive phenols from olive oil by-products. The dissociation of the phenol-β-CD particles for β-CD recycling can be simply achieved by rinsing with ethanol, a food-grade solvent in which the phenols, unlike β-CD, are largely soluble.

Work is under way to apply the process to integral OMWW.

## 3. Experimental

### 3.1. Materials

β-CD was kindly given by Roquettes Frères (Lesterm, France). Folin-Ciocalteu reagent was supplied by Sigma-Aldrich^®^ (Gillingham, UK). Analytical grade methanol and water were from VWR^®^ (Fontenay-sous-Bois, France) and Fisher Scientific^®^ (Leics, UK), respectively. Tyr was supplied by Sigma-Aldrich^®^ (Deisenhofer, Germany) and HT was purchased from Extrasynthèse (Genay, France).

### 3.2. Spectroscopic Analysis

UV-visible analyses were performed on an Agilent^®^ 8453 UV-Visible spectrophotometer (Waldbronn, Germany) in a quartz cell (1 cm path length). For quantification, the molar absorption coefficients of the olive phenols were determined: ε_(Tyr)_ = 1414 M^−1^ cm^−1^ at 275 nm, ε_(HT)_ = 2568 M^−1^ cm^−1^ at 280 nm.

#### 3.2.1. Standard Solution

To optimize the phenol-β-CD molar ratio, 10 mM biophenol solutions were prepared in 25 mL H_2_O. Then, β-CD was added to a β-CD/phenol molar ratio of 1:1; 3:1; 5:1; 10:1. The β-CD/phenol molar ratio of 1.4:1 corresponds to the β-CD limit of solubility (16 g L^−1^ at 20 °C). To optimize the phenolic concentration, 10 mL of a 100 mM phenol solution were mixed with β-CD at 20 °C. The β-CD/phenol molar ratio of 10:1 was investigated with both Tyr and HT. All analyses were performed on fresh standard solutions. Each analysis was repeated three times.

#### 3.2.2. Kinetic Analysis

Standard solutions were mixed with β-CD in the solid state at 20 °C under stirring at 250 rpm. The phenol concentration in the supernatant was monitored by UV/Vis spectroscopy over 3 h. A brief centrifugation step or dilution into water was carried out if necessary.

### 3.3. Analysis of Complexes in the Solid State

#### 3.3.1. Solid Recovery Procedure

At the end of kinetics, the biophenol+β-CD mixture was filtered on a glass filtering funnel (porosity 5). The recovered solid was dried at 35 °C until the mass variation was less than 10%. The solid recovery was determined as a percentage of the initial solid amount (biophenol+β-CD). It was corrected for the solubilized β-CD amount (16 g L^−1^ at 20 °C) to determine the true solid recovery efficiency.

#### 3.3.2. Loading and Extraction Efficiencies

The final powders recovered from the kinetic procedure were analyzed to determine the amount of Tyr or HT retained by solid β-CD. Thus, 50 mg of powder were rinsed with 2 mL MeOH and filtered on a 0.2 µm PTFE syringe. The loading efficiency Qe (mmol/100 g) and the extraction efficiency (EE) were then calculated as follows:

(1) $$Q_{e}=\frac{C_{p}V_{s}}{m}\times 100$$

(2) $$\mathrm{EE}=\frac{C_{p}V_{s}}{m}\times \frac{m_{\mathrm{recovered}\;\mathrm{solid}}}{C_{i}V_{i}}\times 100$$

where C_p_ = phenol concentration in the MeOH fraction, vs. = volume of MeOH (2 mL), C_i_ = initial phenol concentration, V_i_ = initial volume, and m = mass of the rinsed powder.

### 3.4. Photodegradation

Photodegradation experiments were performed using a Suntest CPS+ photoreactor (Atlas Photonics), equipped with a xenon arc lamp and a filter to prevent the transmission of wavelengths below 290 nm. Each sample was packed into a quartz mold of 4 mm in diameter and 3 mm in thickness and was then irradiated for seven days at 765 W/m^2^. The photoreactor temperature was approximately 35 °C.

The color of the samples was measured each day according to the CIELAB color scale, over a white background on a reflection spectrophotometer (CM-3500d, Minolta, Osaka, Japan). The aperture diameter of the measuring port was 3 mm. After color measurement, the CIELAB-based color difference (∆E*ab) was calculated as:

(3) $$∆E_{ab}^{*}=\sqrt{[{\left(∆L^{*}\right)}^{2}+{\left(∆a^{*}\right)}^{2}+\left(∆b^{*}{)}^{2}\right]}$$

### 3.5. Scanning Electron Microscopy

The solid particles obtained at a β-CD/Tyr molar ratio of 10:1 were investigated with a Gemini SEM (Zeiss^®^) scanning electron microscope at a voltage of 3 kV. The microstructure was not coated and was observed at different extensions.

## 4. Conclusions

This patented work describes a one-step procedure for the concomitant removal of olive phenols from wastewater and the formulation of these micronutrients into a native β-CD matrix. It is based on the affinity of solid β-CD for olive phenols.

Extraction efficiencies obtained for Tyr and HT were up to 61%, with a total solid recovery higher than 90%. The UV irradiation of the phenol-β-CD complexes clearly showed the ability of β-CD in the solid state to protect phenols against photodegradation. This procedure is very simple, environmentally friendly (as no organic solvent is involved), and probably much cheaper than the preparation of phenol-β-CD powders by spray- or freeze-drying processes. It could be applied to agricultural wastewaters for the recovery of high-value phenolic compounds for use as food supplements.

## 5. Patents

A patent resulted from the work reported in this manuscript: Tomao, V.; Malapert, M. Method for extracting phenolic compounds. EP 3 603 415 B1, 30 December 2020.

## Figures and Tables

**Table 1:** Solid recovery, extraction, and loading efficiencies for a 10 mM Tyr solution.

	Solid Recovery (%)	Solid Recovery Efficiency (%)	Extraction Efficiency (%)	Loading Efficiency	
				mmol/100 g	mg/g
3:1	22.2 ± 3.9	69.2 ± 3.9	2.2 ± 0.2	2.9 ± 0.3	4.1 ± 0.4
5:1	51.7 ± 4.6	79.9 ± 4.6	6.1 ± 0.4	2.1 ± 0.1	2.9 ± 0.1
10:1	75.4 ± 5.1	89.5 ± 5.1	10.9 ± 1.2	1.3 ± 0.1	1.8 ± 0.1

**Table 2:** Solid recovery, extraction, and loading efficiencies for a 10 mM HT solution.

β-CD/HT Ratio	Solid Recovery (%)	Solid Recovery Efficiency (%)	Extraction Efficiency (%)	Loading Efficiency	
				mmol/100 g	mg/g
3:1	31.4 ± 4.8	78.4 ± 4.8	3.4 ± 0.3	3.2 ± 0.2	4.9 ± 0.3
5:1	57.4 ± 5.4	85.6 ± 5.4	7.1 ± 0.6	2.2 ± 0.0	3.4 ± 0.1
10:1	79.1 ± 3.1	93.2 ± 3.1	13.6 ± 1.3	1.5 ± 0.1	2.3 ± 0.1

**Table 3:** Solid recovery, extraction, and loading efficiencies for 100 mM Tyr or HT solutions.

	β-CD/Phenol Ratio	Solid Recovery (%)	Solid Recovery Efficiency (%)	Extraction Efficiency (%)	Loading Efficiency	
					mmol/100 g	mg/g
Tyr	10:1	91.7 ± 6.2	93.1 ± 6.2	61.9 ± 4.9	5.9 ± 0.1	8.2 ± 0.1
HT	10:1	92.2 ± 3.1	93.6 ± 3.1	61.7 ± 3.1	5.9 ± 0.1	9.1 ± 0.2

## Data Availability

Not applicable.

## Sample Availability

Samples of the phenol-β-CD complexes are available from the authors.
